# Bioactive Properties of Kakadu Plum-Blended Products

**DOI:** 10.3390/molecules28062828

**Published:** 2023-03-21

**Authors:** Yuntao Zhou, Anh Dao Thi Phan, Saleha Akter, Eshetu Mulisa Bobasa, Maral Seididamyeh, Dharini Sivakumar, Yasmina Sultanbawa

**Affiliations:** ARC Industrial Transformation Training Centre for Uniquely Australian Foods, Queensland Alliance for Agriculture and Food Innovation, The University of Queensland, Indooroopilly, QLD 4068, Australia

**Keywords:** Kakadu plum, native fruits, functional foods, antioxidant properties, antimicrobial properties, vitamin C, safety, antidiabetic properties

## Abstract

Kakadu plum (*Terminalia ferdinandiana*), endemic to Australia, is growing in popularity due to its high levels of vitamin C and strong antioxidant properties. In this study, Kakadu plum fruit powder was used as a functional food ingredient with other plant materials to develop value-added products to enhance their nutritional and commercial value. The present study determined the bioactive properties of nine products, including three Kakadu plum fruit powder samples produced from different processing batches and five Kakadu plum-blended products. Vitamin C, the total phenolic content, and the ellagic acid content were determined. Bioactive properties such as antioxidant, antidiabetic, and antimicrobial assays were also performed. Cytotoxicity was tested to obtain more specific product information regarding food safety. Kakadu plum-blended products showed lower cytotoxicity and lower bioactive properties (antioxidant and antibacterial activities) in comparison to Kakadu plum powder. However, overall, most of the bioactive properties were shown to be higher in the blends when compared with the commercial blueberry powder as a benchmark antioxidant product. Therefore, there is great potential for Kakadu plum to contribute to the growing functional food and ingredient markets.

## 1. Introduction

“The term “nutraceutical” refers to substances or tissues derived from food that have health benefits for the human body” [1]. Due to their apparent benefits, nutraceuticals tend to be considered as functional foods rather than medicines [2]. As products that are intended to meet specific diets and preventive health care, nutraceuticals are characterized as “specially designed formulations”. They achieves the purpose of preventing and treating certain diseases while supplementing the daily diet [3]. As consumers’ awareness of healthy living increases, people are gradually avoiding the use of products that contain chemical additives because of the possible risks such as cancer and allergies [4]. This has led to the rapid expansion of the global nutrition and health care products market. The global nutraceuticals market was valued at USD 454.55 billion in 2021 and is expected to grow by 9.0% between 2021 and 2030 [5]. With the outbreak of COVID-19 and the fact that there is still no vaccine that can 100% prevent COVID-19, people’s demand for various nutritional dietary supplements is increasing day by day. Statistics show that the susceptible populations are mainly those with weak immune systems, smokers, and the elderly [6], which further promotes people’s emphasis on the immune system. Consuming functional foods that contain vitamins, antioxidants, etc., strengthens the immune system, which essentially increases resistance to viruses [7]. Therefore, people’s expectations for various nutritional and health products that can enhance the human immune system have further promoted their market and application prospects. Surveys have also shown that immune system support is the main reason for purchasing nutritional supplements, accounting for one in five consumers overall [8].

Kakadu plum (*Terminalia ferdinandiana*) is an endemic plant species that grows in the monsoon tropical climate of northern Australia. This fruit has a long history of use by Indigenous Australians and has important medicinal and nutritional value in Aboriginal cultures. *T. ferdinandiana* is a member of the *Combretaceae* family [9], which includes 20 genera and more than 500 species, and it is widely distributed in tropical and subtropical regions around the world [9]. The genus *Terminalia* contains about 200 species, of which 29 species or subspecies can be found in Australia [10]. Of these, 14 species can be found in the Kimberley of western Australia (WA), 12 in the Northern Territory, and 16 in northern Queensland [10].

Despite its unique phytochemical properties, the Kakadu plum contains a high concentration of ascorbic acid (vitamin C) and was identified as a bush plant nutrient by Aboriginal people [11]. In the first report, Kakadu plum fruit contained 2300–3150 mg of ascorbic acid per 100 g of fresh weight (2.3–3.1%). However, on a dry weight basis in another study, the ascorbic acid levels were recorded as 14,038 ± 701 mg per 100 g (~14%) [12]. The Barbados cherry (or acerola, *Malpighia glabra* L.), native to Brazil, is considered to be the fruit with the highest vitamin C content in the world, with an average vitamin C content of about 1.7% fresh weight [13]. Tropical fruits such as Acerola (*Malpighia emarginata*) and Camu-Camu (*Myrciaria dubia*) are known to have significant levels of vitamin C ranging from 1677 to 2280 mg per 100 g fresh weight (FW) [14]. It is clear that the natural ascorbic acid content in the Kakadu plum is higher than these fruits known for their high ascorbic acid content. In addition, vitamin C is an effective reducing agent and a scavenger for free radicals in biological systems. The hydrogen atoms provided by vitamin C can form stable ascorbic acid free radicals, which can effectively reduce oxidative damage in the daily metabolism of the human body [15].

Kakadu plum fruits were found to contain very high levels of polyphenolic compounds, mainly ellagic acid (EA) and gallic acid. Eshetu et al. reported in 2020 that Kakadu plums can still maintain a high level of ellagic acid content (46.6 mg/g) in freeze-dried form [16]. The content of gallic acid is 5.10 ± 0.03 mg/g DW, which is much higher than that of lemons, oranges, and blueberries [17]. A study by Williams et al. (2014) showed that the ellagic acid content of the Kakadu plum (880.9 mg/100 g) was significantly higher than that of boysenberry (166.4 mg/100 g) on a dry weight basis, even though boysenberry is known to be an ellagic-acid-rich natural fruit [12]. In addition, ellagic acid has also been reported to have anti-inflammatory, anti-cancer, and anti-aging properties, as well as improving symptoms such as Alzheimer’s disease, diabetes, and cardiovascular diseases [18,19]. These compounds, like ascorbic acid, also have very high antioxidant properties and can reduce the risk of many chronic diseases such as cardiovascular disease, stroke, and rheumatoid arthritis [12,20].

In recent years, epidemiological findings have linked phenolic compounds (flavonoids, stilbenes, lignans, tannins, and phenolic acids) in fruits with antioxidant properties [21]. Owing to their structural characteristics (the hydroxyl group attached to the benzene ring), polyphenolic compounds can transfer the hydrogen atoms on the active hydroxyl group to free radicals to achieve the purpose of free radical scavenging [22]. In recent years, studies have divided the methods for evaluating the antioxidant properties of compounds in vitro into two categories: hydrogen atom transfer reaction (HAT) and single electron transfer reaction (SET) [23]. In the HAT reaction, the antioxidant provides a hydrogen atom to the free radical so that the antioxidant itself forms a free radical, while the mechanism of the SET reaction makes the antioxidant provide electrons to the free radical, so that the antioxidant breeds to form a free radical cation [24]. Since these two reactions usually occur together, it is difficult to distinguish them. Therefore, based on the different reaction mechanisms of antioxidants, different methods for the determination of antioxidant capacity have been developed. Examples include DPPH and FRAP for SET and ABTS and ORAC for HAT [25].

The antibacterial activity of the Kakadu plum has long been experimentally proven [26]. Although the exact mechanism of how polyphenols kill or inhibit bacterial growth is unclear, existing reports have demonstrated that the polyphenol-precipitated bacterial cell wall interaction ability varies due to differences in the composition of different bacterial cell walls (Papuc et al., 2017). In addition, polyphenols can also inhibit the formation of bacterial biofilms. Borges et al. have studied the effects of gallic acid and ferulic acid on bacteria such as *Escherichia coli* and *Listeria* and confirmed that these two phenolic compounds inhibit bacterial movement and biological film-forming ability [27].

In this study, Kakadu plum is used as a functional food and ingredient together with other edible plant materials to develop value-added products that can enhance nutritional, health, and commercial value for the food products. Therefore, this study has three objectives. Firstly, it investigates the physicochemical properties, water activity, vitamin C, and ellagic acid of the Kakadu plum and its blended products. This study also determines the bioactive properties (antimicrobial, antioxidant, and anti-diabetic) and compares them to blueberries as a commercial control, and finally, it determines the safety of the Kakadu plum and its blended products.

## 2. Results and Discussion

### 2.1. Water Activity

**Figure 1:**
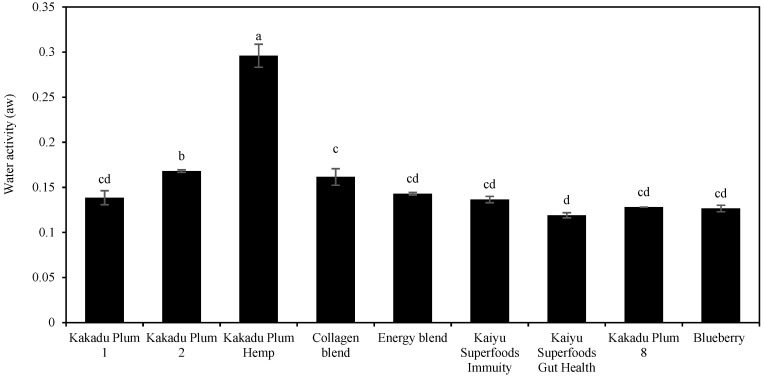
Water activity (aw) of three pure Kakadu plum powder, five Kakadu plum-blended products, and the reference sample blueberry. Data are mean ± standard deviation (*n* = 3); data with different letters are significantly different (*p* ≤ 0.05).

Water activities of three Kakadu plum samples, five Kakadu plum-blended products, and blueberry are shown in Figure 1. The water activity (aw) of all of the samples except Kakadu plum hemp ranged from 0.17 to 0.14. Compared with Kakadu plum hemp, and Kakadu plum 2, all other products showed lower water activity. Compared to the collagen blend, Kakadu plum 2, and Kakadu plum hemp, the Kaiyu Superfoods gut health product (0.119 aw) showed the lowest water activity. The only sample that had a water activity higher than 0.2 was Kakadu plum hemp (0.296), probably due to the high amount of protein in it, which resulted in more bound water [28]. Although higher than the rest of the samples, a water activity of 0.296 is still a low value, indicating that all the samples were dry, safe, and stable (aw < 0.5). This means that the water in the sample is bound to water, which will not affect the shelf life of the food and can also effectively inhibit the growth of microorganisms [29]. However, it also needs to be stored in a dark and dry place, otherwise it will cause the sample to rehydrate.

### 2.2. Vitamin C Analysis

**Figure 2:**
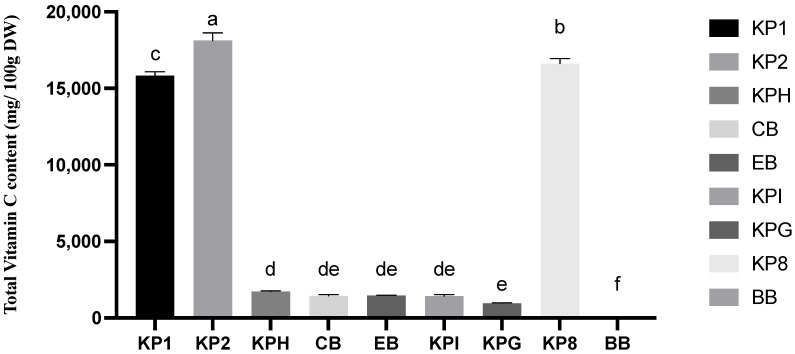
Total vitamin C content (mg/100 g DW) of three pure Kakadu plum powders, five Kakadu plum blend products, and blueberry. Data are mean ± standard deviation (*n* = 3); data with different letters are significantly different (*p* ≤ 0.05).

The vitamin C content of three Kakadu plum powders, five Kakadu plum-blended products, and blueberry is shown in Figure 2 and Table S2. Among the studied samples, the highest vitamin C content was observed in Kakadu plum powder 2 (18,125.85 mg/100 g DW), followed by Kakadu plum powder 8 (16,619.40 mg/100 g DW), and Kakadu plum powder 1 (15,839.51 mg/100 g DW), while the lowest vitamin C was found in Kaiyu Superfoods gut health (959.0 mg/100 g DW). Kakadu plum powder samples 1, 2, and 8, with the fruits harvested from the same batch, have large differences in the content of vitamin C with 15,839.51 mg/100 g DW, 18,125.85 mg/100 g DW, and 16,619.4051 mg/100 g DW, respectively, which could be attributed to many reasons such as harvesting fruits from different locations and not standardized fruit sizes in terms of maturity or different processing conditions during production [30]. The vitamin C content of Kakadu plum hemp, energy blend, collagen blend, and Kaiyu Superfoods immunity samples is not significantly different, but they are all higher than 1400 mg/100 g DW. The Kaiyu Superfoods immunity with Quandong maintained the vitamin C content in comparison to the other tested products such as Kakadu plum hemp, collagen blend, energy blend, Kaiyu Superfoods immunity, and gut health. The observed lowest vitamin C content in the gut health blend, among the five products containing Kakadu plum, may be due to the addition of tropical vegetables and fruits in larger proportions. However, comparing the vitamin C content of the blueberry sample (36.89 mg/100 g DW), the vitamin C of the five products containing Kakadu plum was much higher than that of blueberry. The Australian Government National Health and Medical Research Council recommends a daily intake of 45 mg of vitamin C for a 31–50-year-old adult man [31]. This study further confirms that the five products containing Kakadu plum are very good sources of vitamin C.

### 2.3. Phenolic Content

#### 2.3.1. Total Phenolic Content (TPC)

**Figure 3:**
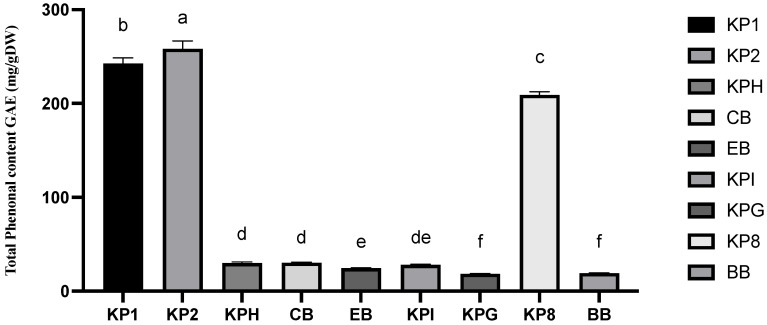
Total phenolic content of three pure Kakadu plum powder, five Kakadu plum-derived products, and blueberry. Data are mean ± standard deviation (*n* = 3); data with different letters are significantly different (*p* ≤ 0.05).

Figure 3 shows the TPC of three Kakadu plum fruit powders, five Kakadu plum-blended products, and blueberry. The highest TPC was found in Kakadu plum powder 2 (258.36 mg/g DW). The TPC content of Kakadu plum powder (samples 1, 2, and 8) was between 200 and 258.36 mg/g DW, while the TPC content of the Kakadu plum-blended product extract was around 30–180 mg/g DW (Table S2). Although there is no significant difference (*p* > 0.05) between the Kakadu plum-blended product such as Kakadu plum hemp, collagen blend, and Kaiyu Superfoods immunity, these products have certain advantages when compared with gut health blend and blueberries. The TPC content of blueberry was 19.3 mg/g DW, while the TPC content of four Kakadu plum-blended product extracts was higher than that of blueberry; data can be seen in Supplementary Materials.

The total phenolic contents of Kakadu plum 1 (242.54 mg GAE/g DW), Kakadu plum 2 (258.36 mg GAE/g DW), and Kakadu plum 8 (209.22 mg GAE/g DW) are significantly different, which could be due to harvesting fruits from different locations and differences in fruit sizes in terms of maturity or different processing conditions applied during production [9].

From the perspective of product ingredients, the main ingredients in the collagen blend are Kakadu plum powder and Faba bean protein powder and other tropical fruits, so the main component that provides phenolic compounds would still be Kakadu plum. It has been reported that Faba beans contain phenolic compounds [32]; it is uncertain whether the slight difference here is due to some phenolic compounds in the isolated Faba bean protein. Additionally, the main ingredients in Kakadu plum hemp are Kakadu plum powder and hemp seed protein powder, which may also explain the similarity of the total phenolic content in these two samples. Comparing the ingredients of Kaiyu Superfoods immunity and energy blend, in addition to the main ingredient of Kakadu plum powder, Quandong and Spirulina are also added in the Kaiyu Superfoods immunity, while the main ingredient in the energy blend is Kakadu plum powder, Spirulina, and River mint. Of the two products, Quandong and River mint both provide phenolic compounds; however, Quandong is known for its antioxidant activity and it may provide more phenolic compounds. The nutritional value of Spirulina lies in its protein and mineral content and it most likely would not affect the total phenolic content. In the gut health blend, the content of Kakadu plum powder is smaller, and the components of tropical fruits and vegetables account for a larger percentage; hence, the phenolic compound in this product is smaller and is comparable to blueberries.

#### 2.3.2. Ellagic Acid

**Figure 4:**
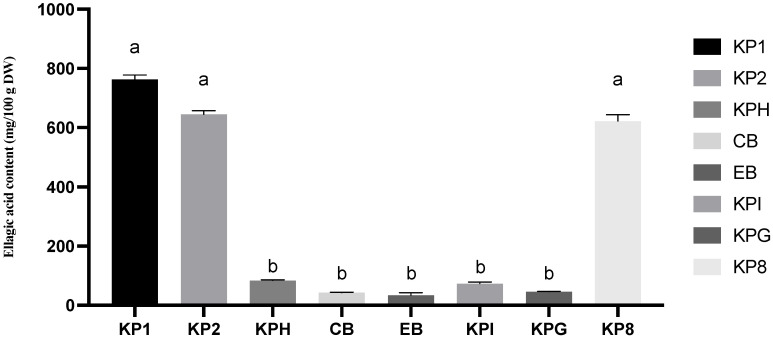
Ellagic acid content of three pure Kakadu plum powders and five Kakadu plum-blended products. Data are mean ± standard deviation (*n* = 3); data with different letters are significantly different (*p* ≤ 0.05).

The ellagic acid contents of three Kakadu plum powders, five Kakadu plum-blended products, and blueberry are presented in Figure 4 and Table S2. Among all the samples, the Kakadu plum powder samples contained the highest total ellagic acid (both free and total ellagic acid). The free ellagic acid content of pure Kakadu plum powder is between 600 and 800 mg/100 g DW, and the total ellagic acid content ranged from 2823 to 2872 mg/100 g DW, which was higher than all other products. The free ellagic acid content of the blended products containing Kakadu plum was below 100 mg/100 g DW, and the total ellagic acid content was about 200 mg/100 g DW.

Among the five products containing Kakadu plum, the total ellagic acid content did not differ significantly. Kakadu plum hemp had the highest free ellagic acid content compared to the collagen blend, energy blend, and gut health blend. In nature, ellagic acid cannot only exist in free form, but more often in a condensed form, such as ellagitannins. Ellagitannins, the esterified form of ellagic acid, are commonly found as constituents of plant cell walls and membranes. Ellagic acid has been shown to be a potent anticancer agent capable of preventing carcinogenic effects caused by certain chemicals by regulating the metabolism of environmental toxins [33].

The benefits of consuming foods that contain more phenolic compounds, known for their role in controlling oxidative stress-related diseases, are clear. In organisms, free radicals cause oxidative damage to nucleic acids, proteins, and fats, leading to the aging of the body and various diseases. The phenolic compounds in plants can act as antioxidants to reduce free radicals due to their phenolic ring and hydroxyl substituents, so as to remove harmful free radicals and inhibit their oxidation reaction with important biomolecules in the body [34].

### 2.4. Bioactivities

#### 2.4.1. Antimicrobial Activity

The antimicrobial activities of Kakadu plum powder and its blended products as well as the commercial blueberry powder were assessed by an agar well diffusion assay and are presented in Supplementary Table S1. Kakadu plum powder and its products exhibited a higher antimicrobial effect compared to the commercial blueberry powder, although the “energy blend” did not show any inhibitory effect against the investigated microorganisms. The Kakadu plum powders showed a higher antimicrobial activity against *S*. *aureus* (20–20.7 mm), MRSA (6–6.9 mm), *B*. *cereus* (12.5–13 mm), and both strains of *P*. *aeruginosa* (4.9–6.8 mm) (Supplementary Table S1). However, the screened samples showed no inhibitory effect against *E*. *coli*, which was in line with the data reported by Bobasa and colleagues [35]. Wright and co-authors reported the inhibitory effect of *T. ferdinandiana* against *Shewanella* spp., which is not in agreement with our results.

The higher inhibition of Gram-positive bacteria than Gram-negative is generally attributed to their membrane structural differences. The monolayer membrane of Gram-positive bacteria facilitates the diffusion of antimicrobial compounds into the microbial cells. However, Gram-negative bacteria possess an outer membrane consisting of hydrophilic polysaccharide, O antigen, and lipid A, which causes more resistance to penetration by antimicrobial compounds into the cell matrix [36]. Furthermore, the Kakadu plum powder and its products did not show any inhibitory effect against the tested fungi. These results are in agreement with those reported previously [37,38]. Generally, fungi are more resistant than bacteria, which is related to their various defense mechanisms such as utilizing phenolic compounds and organic acids as carbon sources and adjusting cell wall conformation to reduce the penetration of antimicrobial agents across their cell walls [39]. Overall, our results showed that blending the Kakadu plum powder with the other ingredients did not improve the antimicrobial activity against the tested food pathogenic microorganisms.

#### 2.4.2. Antioxidant Properties: DPPH Free Radical Scavenging Capacity Assay

**Figure 5:**
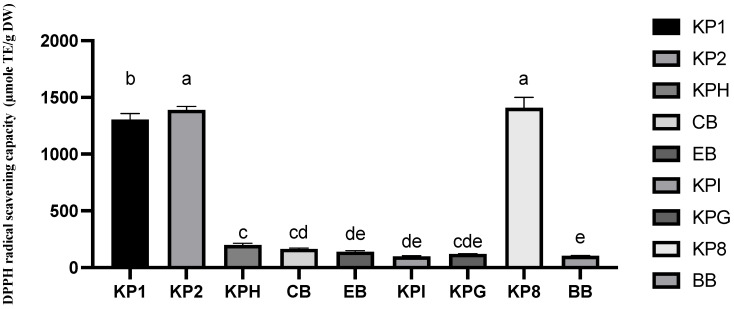
DPPH radical scavenging activity of three pure Kakadu plum powders, five Kakadu plum-blended products, and commercial reference fruit blueberry. Data are mean ± standard deviation (*n* = 3); data with different letters are significantly different (*p* ≤ 0.05).

The DPPH radical scavenging capacity of three Kakadu plum powder samples, five Kakadu plum-blended products, and blueberry are shown in Figure 5 and Table S2. Like the TPC content, the DPPH scavenging activities of Kakadu plum powders (1, 2, and 8) were much higher than other products and ranged from 1300 to 1400 µmole TE/g DW, while the remaining samples of the Kakadu plum blends were around 200–1214 µmole TE/g DW.

Among all the samples, Kakadu plum powder 8 (1408.9 µmole TE/g DW) had the highest DPPH radical scavenging activity. The DPPH scavenging activity of the energy blend, Kaiyu Superfoods immunity, and Kaiyu Superfoods gut health was similar to that of blueberries. Kakadu plum hemp and collagen blend showed a significantly higher DPPH scavenging activity compared to commercial blueberry fruits. The DPPH scavenging activity of Kakadu plum hemp was significantly greater than that of the products pf the energy blend and Kaiya Superfoods immunity. Sharma et al. [40] have reported that the experiments on the DPPH radical scavenging ability of three standard antioxidants show that the scavenging reaction between ascorbic acid and DPPH free radicals is the fastest, and also the strongest. At the same time, the DPPH free radical scavenging ability of ellagic acid is also like that of ascorbic acid [41]. It was also mentioned in the same report that both ellagic acid and ascorbic acid showed a high level of DPPH radical scavenging ability compared to ABTS, superoxide anion radical scavenging ability, and hydrogen peroxide scavenging ability [41].

The relationship between the total phenolic, total ellagic acid, or total ascorbic acid in 8 Kakadu plum blend products and their antioxidant capacity has also been analyzed and found that there is a positive correlation between TPC, vitamin C, and ellagic acid levels. The total ellagic acid content showed the highest correlation with the DPPH scavenging activity (r^2^ = 99.6%), followed by the total ascorbic acid (r^2^ = 99.5%) and total phenolics (r^2^ = 97.7%), which are all very strong correlations. The results suggest that the high levels of ellagic acid and ascorbic acid in the tested samples could be the main contributor to the strong antioxidant properties observed in the studied products. The number of hydroxyl groups present in the molecular structure of ellagic acid and vitamin C can donate electrons/hydrogen atoms to DPPH, a stable free radical reagent, and scavenge large numbers of reactive oxygens. The hydrogen atoms provided by the hydroxyl groups from the bioactive compounds can reverse the reduction oxidation process [42]. Furthermore, a previous study showed that ellagic acid exhibited a strong antioxidant efficacy against lipid peroxidation [43].

#### 2.4.3. Antidiabetic Assay

Table 1 shows the inhibition of α-glucosidase activity by the Kakadu plum and the Kakadu plum-blended products. α-glucosidase, as one of the important carbohydrate-digesting enzymes in the human intestine, exists on the surface membrane of the brush border of enterocytes [44]. With the increasing obesity and aging of the general population worldwide in recent years, the number of patients with hyperglycemic diseases, such as diabetes, is also increasing rapidly. Therefore, methods for controlling postprandial blood glucose levels have received extensive attention. α-glucosidase mainly acts on the absorption of glucose. Blood sugar levels can be controlled by inhibiting α-glucosidase [45].

Among all the tested products and powders of Kakadu plum, all three Kakadu plum powders (1, 2, and 8) and Kakadu plum hemp powder exhibited a strong inhibition on α-glucosidase activity (low IC_50_ values), which was between 0.16 and 0.19 mg/mL. Of the five products containing Kakadu plum, the commercial reference fruit blueberry showed the lowest inhibition of α-glucosidase activity. On the other hand, all three powders of Kakadu plum and five Kakadu plum-blended products showed a higher inhibition of α-glucosidase activity compared to the commercial inhibitor acarbose. The hydroxyl group in the polyphenol structure can inhibit the activity of α-glucosidase, and the carbon–carbon double bond in the phenolic ring also plays a certain role [46]. In our study, the content of polyphenols, ellagic acid, and vitamin C did not show a positive correlation with the α-glucosidase inhibitory capacity. This may be due to the influence of non-phenolic compounds in the samples, as it was also mentioned in another report that foods that are rich in phenolic compounds did not always show a strong inhibitory effect on α-glucosidase [47]. Another report mentioned that compared with large molecular-weight phenolic compounds, smaller molecular-weight phenolic compounds such as catechins are more likely to bind to the active site of the enzyme and thus have a stronger inhibitory effect on α-glucosidase [48]. The molecular weight of hydrolysable tannins in Kakadu plums is mainly between 600 and 1100 g/mol, which belongs to high molecular weight tannins [35]. Therefore, in our results, Kakadu plum-blended products showed a higher inhibitory ability to α-glucosidase than pure Kakadu plum, which may be due to this reason. However, further experiments are required to confirm the exact mechanism.

### 2.5. Cytotoxicity Assessment

**Figure 6:**
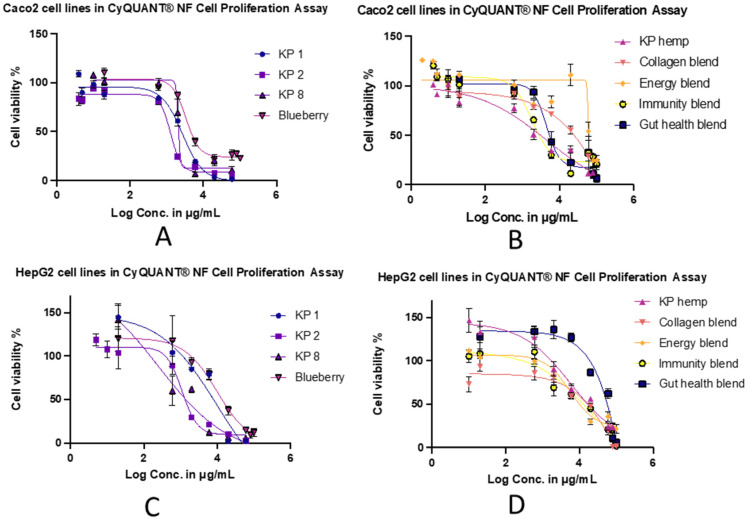
The treatment–concentration response curves of Kakadu plum powders, Kakadu plum-blended products, and blueberry. (**A**,**B**) cell viability with Caco-2 and (**C**,**D**) cell viability with HepG2 cells. Data are presented as the mean percentage of cell viability (±SEM) of three replicates for each treatment.

**Figure 7:**
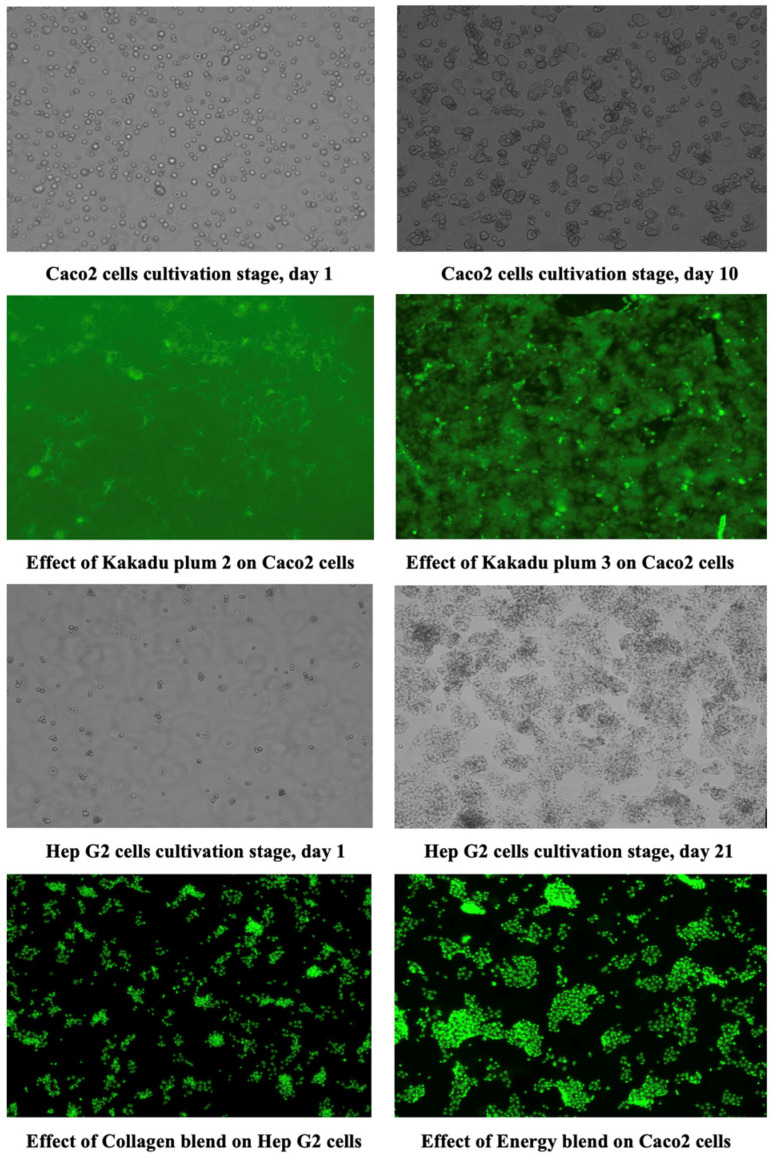
Microscopy images (EVOS M5000) of the Caco-2 and HepG2 cells during cell growth and after treatment with the extracts of Kakadu plum products.

A CyQUANT^®^ NF Cell Proliferation Assay kit was used to determine the cell viability after treatment with the extracts of Kakadu plum powder, Kakadu plum-blended products, and blueberry. The extract of each product reduced the cell viability of the tested cell lines in a concentration-dependent manner. The treatment–concentration response curves are shown in Figure 6. The IC_50_ values calculated from these curves are listed in Table 2 Microscopy images (EVOS M5000, Thermo Fisher Scientific, Singapore) of the Caco-2 and HepG2 cells during cell growth and after treatment with the extracts of Kakadu plum powder and Kakadu plum-blended products are presented in Figure 7.

An in vitro assessment of the cell viability of a new product is important to promote the potential safety of the product [49]. In an in vitro cell viability assay, the effect of the tested compounds on the viability of cells grown in a culture is determined by measuring the number of viable cells remaining after an incubation period [50]. By comparing the effects of the extracts of different products on the viability of both cell types, it was found that the cell viability of HepG2 was impacted more than the Caco-2 cells. Kakadu plum-blended products had less impact on the viability of both cells compared to pure Kakadu plum powders. In Caco-2 cells, the IC_50_ value obtained from the blueberry sample was like Kakadu plum pure powder (sample 1). In HepG2 cells, the blueberry sample was more cytotoxic than the Kakadu plum pure powder (sample 1). Energy blend, collagen blend, and KP hemp were found to be safer than the other samples in both cells. Kakadu plum pure powder (sample 2) was found to be the most active sample in reducing cell viability in HepG2 cells. Blueberry and immunity blend were found to be the most active samples in reducing the cell viability in Caco-2 cells. Among the three pure Kakadu plum products, Kakadu plum 1 and 8 had a greater effect on the cell viability of both cell lines. Among the five Kakadu plum-blended products, the immunity blend had the greatest impact on the cell viability of Caco-2, which was also greater than the blueberry product. Kakadu plum hemp and the energy blend had the greatest impact on the cell viability of HepG2, which was also greater than that of blueberry. Overall, the five Kakadu plum-blended products had less impact on the cell viability than pure Kakadu plum powders and blueberries, which means that the cytotoxicity of the five Kakadu plum-blended products was lower than pure Kakadu plum powder and commercial blueberry products. Kakadu plum powders have been reported to contain higher amounts of bioactive polyphenolic compounds, which might be the reason for reducing the cell viability. Moreover, the Kakadu plum-blended products are composed of various other ingredients besides Kakadu plum, so the concentrations of the bioactive polyphenolic compounds would be lower in those products compared to pure Kakadu plum powders. Although enterocyte assays serve as one of the most common in vitro techniques for simulating intestinal absorption, cell-based assays cannot fully replace in vivo studies, as these models do not fully reflect all human cells, tissues, and biological functions [50], so the results can be used as a reference to design future in vivo experiments to determine the safety profile of Kakadu plum products.

## 3. Materials and Methods

### 3.1. Materials

#### 3.1.1. Chemicals and Reagents

Gallic acid, ascorbic acid, and ellagic acid (>95% purity) were sourced from Sigma Aldrich (Sydney, NSW, Australia). DPPH [2,2-diphenyl-1-picryl hydrazyl], acarbose, sodium carbonate, metaphosphoric acid, acetic acid, ethylenediaminetetraacetic acid (EDTA), formic acid, hydrochloric acid, *p-*nitrophenyl-α-d-glucopyranoside (pNPG), α-glucosidase, acarbose, organic solvents (HPLC-grade), and other reagents used throughout the study were supplied by Merck or Sigma Aldrich (Sydney, NSW, Australia). In the cytotoxicity assessment, the Caco-2 cell line was purchased from the American Type Culture Collection (Manassas, VA, USA), and HepG2 cell lines were purchased from Sigma Aldrich (Sydney, NSW, Australia). Nunc cell culture flasks and 96-well plates were purchased from Sigma-Aldrich (Sydney, NSW, Australia). Hank’s Balanced Salt Solution (HBSS), penicillin and streptomycin, fetal bovine serum (FBS), glutamate, trypsin-EDTA, non-essential amino acids (NEAA), Dulbecco’s modified eagle medium (DMEM), Dulbecco’s phosphate-buffered saline without calcium and magnesium (PBS), trypan blue exclusion dye, and CyQUANT^®^ NF Cell Proliferation Assay reagent (Molecular Probes™) were purchased from Invitrogen (Thermo Fisher Scientific Corporation, Waltham, MA, USA).

#### 3.1.2. Test Microorganisms

*Staphylococcus aureus* (NCTC 6571) and *Escherichia coli* (NCTC 9001) were supplied by the National Collection of Type Cultures (NCTC, Health Protection Agency Center for Infection, London, UK). Methicillin-Resistant *Staphylococcus aureus* (clinical isolate), *Bacillus cereus* (ATCC 10876), *Shewanella putrefaciens* (ATCC 8071), *Pseudomonas aeruginosa* (ATCC 10145), *Pseudomonas aeruginosa* (clinical isolate), *Aspergillus flavus* (ATCC 26944), and *Candida albicans* (ATCC 10231) were sourced from the American Type Culture Collection (ATCC, In Vitro Technologies Pty, Ltd., Melbourne, Australia).

#### 3.1.3. Samples

Kaiyu Superfoods is a First Nations-owned and led company producing a range of healthy products containing Kakadu plum fruit powder. In the project, Kakadu plum fruit powder products listed in Table 3 were sourced from Kaiyu Superfoods (Darwin, Northern Territory) and stored at −40 °C. Kaiyu Superfoods samples included collagen blend, energy blend, immunity blend, and gut health blend. The blueberry powder was purchased from matcha leaf superfoods in NSW, Australia.

### 3.2. Water Activity

The water activity of the powdered samples was determined according to AOAC method 934.01, using a LabTouch-aw water activity meter (Novasina AG, Lachen, Switzerland). Briefly, 2 g of powdered samples was placed evenly into a standard measuring cup and incubated at a constant temperature of 25 ± 1 °C for 10 min before the aw measurement. The measurements were conducted in duplicates.

### 3.3. Determination of Vitamin C

Extraction and analysis of vitamin C followed the method reported previously [11], which had been adapted from the method published previously [51]. Briefly, approximately 200 mg of powdered samples was extracted with a 3% metaphosphoric acid solution containing 8% acetic acid and 1 mM ethylenediaminetetraacetic acid (EDTA). The dehydro-ascorbic acid that was present in the extract was converted to ascorbic acid using 40 mM Dithiothreitol (DTT) at pH 9.0 before HPLC-PDA analysis, with a Vanquish HPLC-PDA system (Thermo Fisher Scientific, Brisbane, Australia). A reverse-phase Waters^®^ HSS-T3 column (150 × 2.1 mm i.d; 1.8 µm, Waters, Sydney, NSW, Australia) maintained at 25 °C was used to separate the compound with an isocratic eluent at a flow rate of 0.25 mL/min with 0.1% formic acid in Milli-Q water. The external calibration curve of the ascorbic acid standard (0–192.5 μg/mL, y = 0.4481x − 0.0044, r^2^ = 0.9963, and LOD = 0.5 ppm) was used to quantify the vitamin C content. The extraction was conducted in triplicates.

### 3.4. Determination of Total Phenolic Content (TPC) and DPPH Free Radical Scavenging Activity

Total phenolic compounds were extracted using an 80% (*v*/*v*) acidified methanol solution containing 0.1% concentrated HCl. The fruit powder sample (0.5 g) was homogenized with 80% acidified methanol and vortexed for 30 s and thereafter, sonicated in an ultrasonic bath for 15 min (Soniclean, Model 500HD, SA, Australia). After centrifugation at 4000 rpm for 10 min (Multifuge X Pro Series, Centrifuge, Thermo Fisher Scientific, Waltham, MA, USA), the upper layer of clear liquid was retained. The residue was re-extracted twice, while the supernatant was collected. Five mL of extractant was added to the collected supernatant to a final volume of twenty mL of the extraction solution.

The TPC was determined using a Folin–Ciocalteu assay [52], using a microplate absorbance reader (Varioskan LUX, Thermo Fisher Scientific, Singapore) monitored at 700 nm. The TPC was equivalently quantified based on the external curve of gallic acid standard (0–120 μg/mL prepared in milli-Q water). The assay was performed in triplicates, and the results were expressed as gallic acid equivalent per gram of dry weight (mg GAE /100 g DW) [35].

The total antioxidant activity was determined using DPPH free radical scavenging capacity assay [53] using a microplate absorbance reader (Varioskan LUX) and measuring the absorbance at 517 nm. The experiment was performed in triplicates and the DPPH free radical scavenging ability was expressed as μM Trolox equivalent per g sample in dry weight. A Trolox external standard calibration curve was prepared from 5 to 35 μM/mL.

### 3.5. Determination of Ellagic Acid

Extraction and analysis of ellagic acid followed the method previously described by Williams et al. with slight modifications [54]. Approximately 200 mg powdered samples were extracted with 80% (*v*/*v*) methanol containing 0.01N HCl to release both free and conjugated ellagic acids. Next, the ellagic acid existing as an esterified form in the above extract was acid hydrolyzed overnight at 90 °C into a 5 mL Reacti-Therm vial (Thermo Fisher Scientific, Brisbane, Australia) using 2N HCl, as previously described previously [54] without any modifications. Ellagic acid released after the hydrolysis was re-dissolved into absolute methanol and injected into a Vanquish HPLC-PDA system (Thermo Fisher Scientific, Brisbane, Australia) for quantification of total ellagic acid content (both free and conjugated forms). The extraction and hydrolysis were conducted in triplicates. A Waters^®^ BEH Shield RP C18 column (100 mm × 2.1 mm i.d;1.7 μm, Waters, Sydney, NSW, Australia) maintained at 40 °C was used to separate the compound using a solvent gradient program at the flow rate of 0.3 mL/min, consisting of 0.1% formic acid in milli-Q water (mobile phase A), and 0.1% formic acid in acetonitrile (mobile phase B). Gradient elution programmed for mobile phase B was as follows: 5%: 1 min, 20%: 4 min, 40%: 10 min, and 95%: 2 min, and re-equilibration for 5 min before the next injection. Total ellagic acid was quantified using an external calibration curve of ellagic acid monitored at 254 nm (ranged from 0 to 118.5 μg/mL, y = 0.285x − 0.7345, r^2^ = 0.9944, and LOD = 1 ppm). Representative HPLC-PDA chromatograms and UV spectra (scanned from 200 to 400 nm) of the extracts, ellagic acid standard, and the blank (MeOH) are present in Supplementary Figures S1 and S2, respectively.

### 3.6. Antimicrobial Assay

Agar well-diffusion assay was used for screening the antimicrobial activity of the samples [55] against selected food-related microorganisms. Samples (ca. 0.5 g) were mixed with 80% methanol, followed by vortexing (Thermo Fisher Scientific, Waltham, MA, USA), 15 min sonication (Soniclean, Model 500HD, SA, Australia), and centrifugation (Multifuge X Pro Series, Centrifuge, Thermo Fisher Scientific, Waltham, MA, USA) at 4000 rpm for 10 min. The supernatant was collected, and the pellet was re-extracted twice. The combined supernatants were evaporated under nitrogen flow, which was followed by freeze-drying (−50 °C, 0.04 mbar). The extract solutions (60 mg/mL) were prepared in 20% aqueous dimethyl sulfoxide (DMSO). Streptomycin (20 μg/mL) was used as the positive control and 20% DMSO as a negative control. 

Briefly, the inoculums (10^6^ CFU/mL) of overnight-grown bacteria and yeast and 10-day-old mold were spread on Mueller Hinton (for bacteria) and potato dextrose (for fungi) agar plates. Eight-millimeter wells were punched into the plate and filled with 100 µL of the sample. Plates were then incubated at 25 °C for 48 h (fungi) and 30 °C for 24 h (bacteria). The inhibition zone diameter (mm) was measured by a digital caliper (±0.01 mm) and subtracted from the well diameter. The sensitivity according to the diameter of the inhibition zone can be categorized as <8 mm not sensitive, 9–14 mm sensitive, 15–19 mm very sensitive, and >20 mm extremely sensitive [56]. All experiments were carried out in triplicates, and results are expressed as mean ± standard deviation (SD). We calculated basic variance statistics for the obtained data using the statistical program Graphpad Prism v 9.4.1 (San Diego, CA, USA).

### 3.7. Cytotoxicity Assessment

The human Caco-2 cell line derived from colon carcinoma is the most common cell line that is widely used as an in vitro model of the intestinal epithelial barrier, and the human HepG2 cell line derived from a hepatocellular carcinoma is widely used as an in vitro model of human liver epithelial cells. In the current study, all the Kakadu plum products and blended products are hypothesized to be rich source of polyphenols. Therefore, potential cytotoxicity of the polyphenols present in the products will be explored in the intestine and liver cell lines [50].

Caco-2 cells were cultured using DMEM supplemented with 10% FBS (*v*/*v*), 1X NEAA, penicillin 100 U/mL, streptomycin 100 μg/mL, and Glutamax 2 mM and HepG2 cells were maintained in DMEM supplemented with 10% FBS (*v*/*v*, penicillin 100 U/mL, streptomycin 100 μg/mL, and Glutamax 2 mM). Cells were grown in vented culture flasks at 37 °C and 5% CO_2_. Cells were passaged at 90% confluency every 2–3 days. Sub-culturing of cells was performed following the same protocol described earlier by Akter and colleagues [50]. Cells were counted using trypan blue exclusion staining with the cell suspension appropriately diluted for cell passage using Countess 3 automated cell counter (Invitrogen, Thermo Fisher Scientific Corporation, Waltham, MA, USA).

CyQUANT^®^ NF Cell Proliferation Assay was used to determine the cell viability upon treatment with the extracts, according to the method described by Akter and co-authors [50]. Dry extract pellets of the samples were used for the cell viability assay to determine the safety of the extracts. For the Caco-2 cell model, 4 × 10^4^ cells/well and for the HepG2 cell model, 5 × 10^4^ cells/well (100 μL) were plated in 96-well plates and incubated at 37 °C and 5% CO_2_ for 24 h. A growth medium (100 μL) without cells was added to the control wells to obtain a value for background fluorescence. On the experiment day, the culture media was carefully removed and replaced with 100 μL HBSS and kept incubating for 2 h at 37 °C and 5% CO_2_. After 2 h, HBSS was carefully removed and replaced with 50 μL sample solutions. For the control wells, 50 μL HBSS was added. The plates were incubated for 2 h at 37 °C and 5% CO_2_ and after 2 h, the sample solution was removed, and 100 μL HBSS was used to wash the cells. Then, the HBSS was removed and 74 μL (1×) dye-binding solutions were added to each well (including the control well) using a manual multichannel pipette. After incubating the plates at 37 °C for an hour, the plates were read to measure the fluorescence with excitation at 485 nm and emission at 530 nm using a microplate reader (Varioskan LUX, Thermo Fisher Scientific, Singapore) [50].

### 3.8. Antidiabetic Assay

The antidiabetic capacity of samples was measured by the inhibition of α-glucosidase activity followed the method described by Zhang et al. [57]. The α-glucosidase activity was determined by measuring the yellow-colored para-nitrophenol released from *p*-nitrophenyl-α-d-glucopyranoside (pNPG) at 405 nm using a microplate reader (Thermoscientific Spectrophotometer, Varioskan LUX, Singapore). The following solutions were prepared for the assay: α-glucosidase solution (11 unit/mg) dissolved in 0.1 M phosphate buffer (pH 6.9) to the concentration of 0.1 mg/mL (1 unit/mL) and 2 mM *p*-nitrophenyl-α-d-glucopyranoside (pNPG) solution (0.0301 g of *p*-nitrophenyl-α-d-glucopyranoside dissolved in 50 mL of 0.1 M phosphate buffer (pH 6.9)). Acarbose (Bayer Pharmaceuticals, Leverkusen, Germany) standard solution was prepared in 1% (*v*/*v*) DMSO and further diluted with Milli-Q water to different concentrations (0.5, 0.25, 0.125, and 0.0625 mg/mL) and used as positive controls. The α-glucosidase inhibitory activities were calculated using the following equation:

Percent inhibition = (Ac − Ae/Ac) × 100

Ac = Absorbance of control; Ae = absorbance of extract/standard.

## 4. Conclusions

Kakadu plum powder shows a higher antioxidant activity, total phenolic content, vitamin C and ellagic acid content, and antibacterial activity in comparison to Kakadu plum-blended products. However, all the Kakadu plum-blended products have a higher antioxidant activity, total phenolic content, vitamin C and ellagic acid content, and antibacterial activity than the blueberry products that were used as a comparison. In terms of cytotoxicity, Kakadu plum-blended products are safer than Kakadu plum powder as the latter contains high levels of bioactive compounds. Other native Australian fruit ingredients in these Kakadu plum-blended products also bring some benefits to the products. For example, products containing Quandong have higher total phenolic and vitamin C contents. Future research on the mechanism of action for antioxidant properties of Kakadu plum powder and the blended products should be investigated. For consumers, we recommend that children, middle-aged, and elderly people choose Kakadu plum-blended products. The cytotoxicity of pure Kakadu plum powder is relatively higher; however, other indicators of Kakadu plum-blended products, such as the total phenolic content and vitamin C content, are still very high compared to blueberries, so it can meet the human body’s daily needs. Other available ingredients added to Kakadu plum-blended products can provide a more diversified nutritional value.

## Figures and Tables

**Table 1:** IC_50_ values obtained in α-glucosidase inhibitory activity assay.

Samples	IC_50_ of α-Glucosidase (mg/mL)
Acarbose (positive control)	1.11 ± 0.09
Kakadu plum 1	0.18 ± 0.001 ^a^
Kakadu plum 2	0.16 ± 0.004 ^a^
Kakadu plum hemp	0.19 ± 0.015 ^ad^
Collagen blend	1.43 ± 0.03 ^b^
Energy blend	0.97 ± 0.002 ^c^
Immunity blend	0.28 ± 0.007 ^d^
Gut health blend	0.43 ± 0.008 ^e^
Kakadu plum 8	0.17 ± 0.007 ^a^
Blueberry	2.15 ± 0.03 ^b^

Results are expressed as mean ± SD (*n* = 6); data with different letters are significantly different (*p* ≤ 0.05).

**Table 2:** IC_50_ values obtained in CyQUANT^®^ NF Cell Proliferation Assay (mg/mL).

	Caco-2	HepG2
KP 1	6.2 ± 1.8 ^a^	8.5 ± 1.3 ^a^
KP 2	13.3 ± 6.0 ^ab^	1.1 ± 0.006 ^a^
KP hemp	23.2 ± 5.0 ^b^	7.8 ± 2.6 ^a^
Collagen blend	23.4 ± 4.0 ^b^	63.5 ± 0.8 ^b^
Energy blend	59.8 ± 1.3 ^c^	63.1 ± 58.1 ^b^
Immunity blend	5.4 ± 0.1 ^a^	64.1 ± 7.5 ^b^
Gut health blend	64.9 ± 8.0 ^c^	58.4 ± 1.1 ^b^
KP 8	1.8 ± 0.6 ^a^	2.5 ± 1.1 ^b^
Blueberry	5.2 ± 4.3 ^a^	55.4 ± 4.5 ^b^

Data are mean ± standard deviation (*n* = 3); data with different letters in the same column are significantly different (*p* ≤ 0.05).

**Table 3:** List of samples studied and their ingredients.

NO	Products	Abbreviation	Ingredients
1	Kakadu plum	KP1	Pure Kakadu plum powder, without seeds, and produced from 2 different processing batches.
2	Kakadu plum	KP2	
3	Kakadu plum hemp	KPH	Kakadu plum powder (without seeds and produced at the same batch with sample 8) and hemp protein powder.
4	Collagen blend	CB	Kakadu plum powder (without seeds and produced at the same batch with sample 8), Faba bean protein powder, and other tropical fruits.
5	Energy blend	EB	Kakadu plum powder (without seeds and produced at the same batch with sample 8), Spirulina, River mint, Faba bean powder, and other tropical fruits.
6	Kaiyu Superfoods immunity	KPI	Kakadu plum powder (without seeds and produced at the same batch with sample 8), Quandong, Spirulina, and other tropical fruits and vegetables.
7	Kaiyu Superfoods gut health	KPG	Kakadu plum powder (without seeds and produced at the same batch with sample 8) and other tropical fruits and vegetables.
8	Kakadu plum	KP8	Pure Kakadu plum powder, without seeds, and produced at different batch with sample 1 and 2.
9	Blueberry	BB	Commercial blueberry powder purchased from matcha leaf superfoods, Australia.

## Data Availability

Data will be available upon request to the corresponding author.

## Supplementary Materials

The following supporting information can be downloaded at: https://www.mdpi.com/article/10.3390/molecules28062828/s1, Table S1: inhibitory effect of Kakadu plum powder and its blend products against several pathogenic microorganisms; Table S2: data of total vitamin C content, total phenolic content, total ellagic acid content, and DPPH scavenging capacity; Figure S1 shows representative HPLC-PDA chromatograms at 254 nm of the extracts before and after acid hydrolysis, ellagic acid standard, and MeOH as a blank; Figure S2 presents the UV spectra scanned from 200 to 400 nm of the standard (top) and the analyte (bottom).
