# Supplementary material for: Bioactive Properties of Kakadu Plum-Blended Products
Source: Molecules. 2023 Mar 21;28(6):2828. doi: 10.3390/molecules28062828 (PMC10056586; doi:10.3390/molecules28062828)
Supplement: Supplementary file 1 [file molecules-28-02828-s001.zip › molecules-2270262-supplementary.pdf]

Supplementary Table S1. Inhibitory effect of Kakadu plum powder and its blend products against several pathogenic microorganisms.

Microorganisms	Zones of inhibition (mm)									
	Kakadu plum 1	Kakadu plum 2	Kakadu plum hemp	Collagen blend	Energy blend	Immunity blend	Gut health blend	Kakadu plum 8	Blueberry	Streptomycin sulfate
S. aureus	20.5±0.4 a	20.7±0.6 a	18.2±0.4 b	-	-	10.9±0.1 c	8.9±0.5 d	20.0±0.4 a	-	15.8±0.03 e
MRSA	6.5±0.2 a	6.9±0.5 ab	-	-	-	-	-	6.0±0.2 a	-	9.02±0.1 c
B. cereus	12.5±0.4 a	13.1±0.2 a	7.0±0.2 b	4.1±0.2 cd	-	4.6±0.3 c	3.3±0.1 d	13.0±0.6 a	1.7±0.3 e	17.71±0.4 f
E. coli	-	-	-	-	-	-	-	-	-	13.48±0.3 a
S. putrefaciens	-	-	-	-	-	-	-	-	-	17.33±0.1 a
P. aeruginosa (clinical isolate)	4.9±0.5 a	6.6±0.5 b	2.7±0.5 c	-	-	-	-	6.2±0.1 b	-	12.77±0.4 d
P. aeruginosa	6.8±0.2 a	5.9±0.3 abc	-	-	-	-	-	5.5±0.1 bc	-	4.79±0.8 c
C. albicans	-	-	-	-	-	-	-	-	-	-
A. flavus	-	-	-	-	-	-	-	-	-	-

Data are mean ± standard deviation (n=3); data with different letters in the same row are significantly different ($p \leq 0.05$). The absence of inhibitory activity is shown by (-). MRSA, methicillin-resistant *Staphylococcus aureus*.

Supplementary Table S2. Data of total vitamin C content, total phenolic content, total ellagic acid content and DPPH scavenging capacity.

	Total Vitamin C content (mg/100g DW)	Total phenolic content (mg GAE/g DW)	Total ellagic acid content (mg/100g DW)	DPPH (µmole TE/g DW)
Kakadu Plum	15839.51±248.14 c	30.00±1.03 b	2870±134.48 a	1306.2±21.23 b
Kakadu Plum	18125.85±493.88 a	30.24±0.69 a	2823±67.63 a	1390.54±12.24 a
Kakadu Plum Hemp	1727.61±50.65 d	24.62±0.45 d	279±3.75 b	199.998±6.17 c
Collagen blend	1446.65±82.25 de	242.54±6.12 d	209±7.74 b	163.587±3.91 cd
Energy blend	1467.79±22.57 de	258.36±8.27 e	177±18.07 b	139.663±2.89 de
Kaiyu Superfoods Immunity	1436.87±81.06 de	209.22±3.32 de	241±14.02 b	99.6386±2.67 de
Kaiyu Superfoods Gut health	959.03±33.20 e	28.25±0.49 f	173±16.16 b	121.387±0.44 cde
Kakadu Plum	16619.4±320.85 b	18.65±0.39 c	2872±106.86 a	1408.99±37.81 a
Blueberry	36.89±1.10 f	19.30±0.26 f		104.116±1.05 e

Data are mean ± standard deviation (n=3); data with different letters in the same column are significantly different ($p \leq 0.05$).

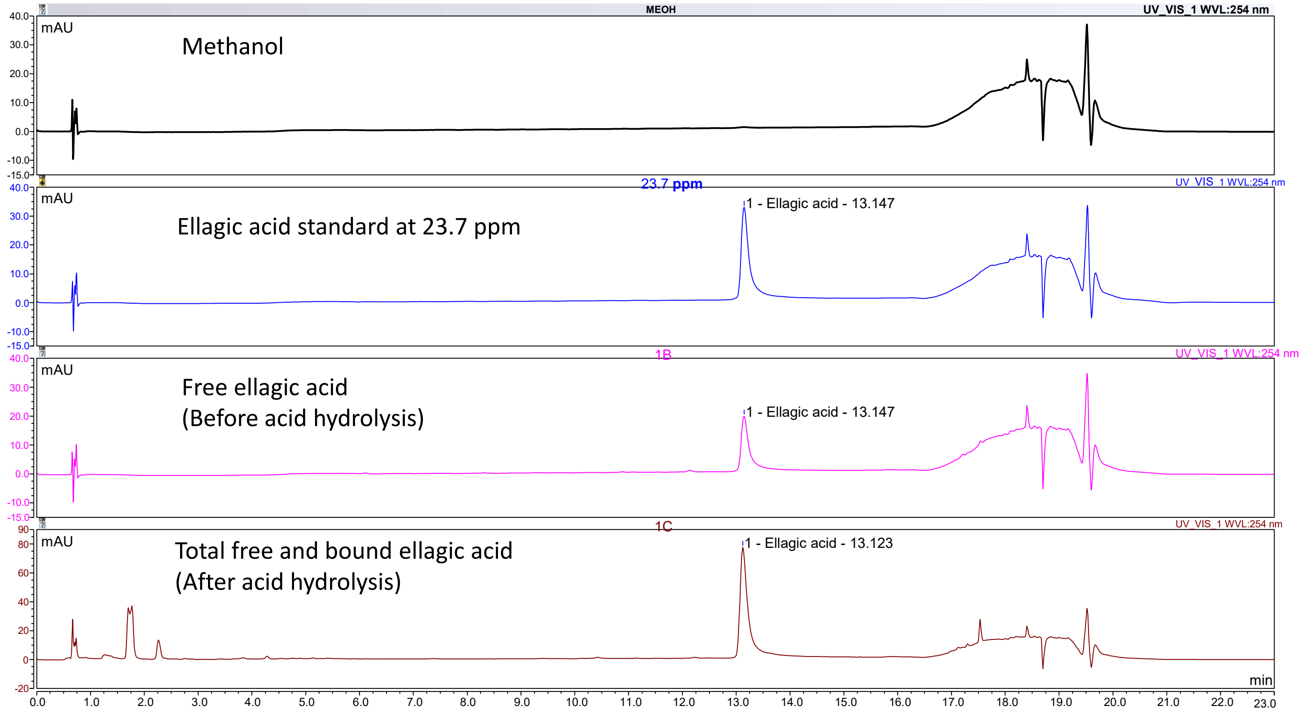

Supplementary Figure S1. Representative HPLC-PDA chromatograms at 254 nm of the extracts before and after acid hydrolysis, ellagic acid standard, and methanol as a blank.

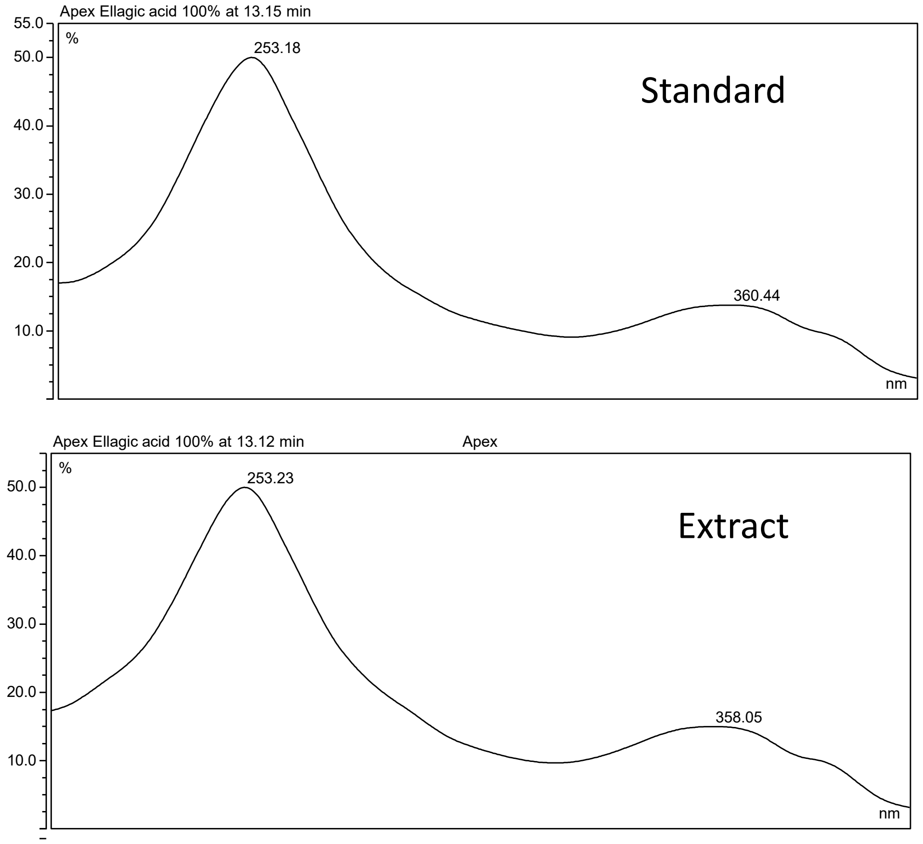

Supplementary Figure S2. The UV spectra scanned from 200 nm to 400 nm of the standard (top) and the analyte (bottom).
